# A Battery-Aware Sensor Fusion Strategy: Unifying Magnetic-Inertial Attitude and Power for Energy-Constrained Motion Systems

**DOI:** 10.3390/s26030856

**Published:** 2026-01-28

**Authors:** Raphael Diego Comesanha e Silva, Thiago Martins, João Paulo Bedretchuk, Victor Noster Kürschner, Anderson Wedderhoff Spengler

**Affiliations:** Electrical Engineering Department, Federal University of Santa Catarina, Florianópolis 88040-900, SC, Brazil; t.m.thiago.martins@posgrad.ufsc.br (T.M.); joao.bedretchuk@posgrad.ufsc.br (J.P.B.); victor.noster@t2f.ufsc.br (V.N.K.); anderson.spengler@ufsc.br (A.W.S.)

**Keywords:** energy-aware orientation, state of charge estimation, efficient consumption, Kalman filter coupling, power-aware motion monitoring

## Abstract

Extended Kalman Filters (EKFs) are widely employed for attitude estimation using Magnetic and Inertial Measurement Units (MIMUs) in battery-powered sensing systems. In such applications, energy availability influences system operation, yet battery state information is commonly treated by external supervisory mechanisms rather than being integrated into the estimation process. This work presents an EKF-based formulation in which the battery State of Charge (SOC) is explicitly included as a state variable, allowing joint estimation of attitude and energy state within a single filtering framework. SOC dynamics are modeled using a low-complexity estimator based on terminal voltage and current measurements, while attitude estimation is performed using a Simplified Extended Kalman Filter (SEKF) tailored for embedded MIMU-based applications. The proposed approach was evaluated through numerical simulations under constant and time-varying load profiles representative of low-power electronic devices. The results indicate that the inclusion of SOC estimation does not affect the attitude estimation performance of the original SEKF, while SOC estimation errors remain below 8% for the evaluated load conditions with power consumption of approximately 0.1 W, consistent with wearable and small autonomous electronic platforms. By incorporating energy state estimation directly into the filtering structure, rather than treating it as an external supervisory task, the proposed formulation offers a unified estimation approach suitable for embedded MIMU-based systems with limited computational and energy resources.

## 1. Introduction

Magnetic and Inertial Measurement Units (MIMUs) are essential for estimating the attitude and orientation in motion monitoring through the fusion of data from accelerometers, gyroscopes, and magnetometers. These sensors are widely used in autonomous systems, wearable devices [1], mobile robotics [2], and indoor/outdoor navigation platforms [3] that operate under strict energy constraints. A standard algorithm for such sensor fusion is the Extended Kalman Filter (EKF) [4,5,6], which enables robust estimation of orientation represented by quaternions or Euler angles.

Despite the maturity of these estimation techniques, energy autonomy remains a major challenge.Battery-powered embedded systems, particularly those employing Micro-Electro-Mechanical Systems (MEMSs) inertial sensors, face severe limitations in long-term operation due to the fundamental trade-off between the accuracy of parameters sensed by the MEMS and the overall system power consumption [4,7]. As system complexity and sensor sampling rates increase, energy demand rises proportionally. This increased consumption is inherent to the execution of complex code and to elevated data acquisition activity, directly impacting the operational lifetime system and potentially affecting system reliability through voltage drops or partial system failures under low-energy conditions. This work targets resource-constrained inertial-magnetic sensing platforms, such as wearable motion-tracking devices and small autonomous systems, where computational power, energy budget, and sensor redundancy are existential and quantitative, aiming to extend a severely limited capacity to the maximum extent, where the microcosmic efficiency of the sensor fusion algorithm is a primordial survival factor for the device.

Battery Management Systems (BMSs), despite requiring considerable processing resources and consuming a significant energy, owing to the added dedicated hardware, computational overhead, and increased system payload [8], have been developed to monitor and estimate battery health and performance. Among the various BMS functionalities, State of Charge (SOC) estimation stands out as a key metric for determining the available energy in real time, supporting power-aware decision-making—that is, the implementation of adaptive strategies focused on energy consumption such as dynamic sensor reconfiguration, task prioritization, and user notifications—and ensuring safe operation [9]. Methods of SOC estimation typically rely on electrochemical models [10], equivalent circuit models [11], or data-driven approaches [12,13]. When integrated with state-estimation frameworks, these models can accurately predict battery capacity, lifetime, degradation, and other parameters essential for energy assessment [14].

It is important to note that the proposed framework is not intended to replace full-featured Battery Management Systems, which provide essential functions such as cell balancing, thermal protection, and safety monitoring. Instead, the proposed method targets applications where real-time SOC awareness is sufficient to enable energy-aware estimation and operation.

Motion monitoring techniques focused on energy efficiency, such as those discussed in the literature presented below, reflect a growing effort to balance attitude estimation and consumption. Solutions range from the use of low-power sensors to hybrid approaches that combine data from multiple internal and external sources [15]. Some methods leverage environmental information, such as nearby Wi-Fi networks or cellular signals, which, although less accurate than GPS, significantly reduce power consumption [16]. More advanced strategies employ accelerometers to enhance energy efficiency [7,17] and magnetometers to improve trajectory tracking by a factor of three or more [18].

Considering the significant energy consumption of sensors in wearable motion-tracking devices and small autonomous systems, several techniques have been investigated to balance energy usage and estimation accuracy. These are classified in Refs. [7,17,18,19,20] as:

- **Duty Cycling:** Dynamically changing sensor operation modes according to a specific context;
- **Sampling Rate Adjustment:** Adapting sensor sampling frequency to balance data quality and consumption;
- **Sensor Selection:** Alternating between sensors of different types and power profiles depending on operating conditions;
- **Clustering:** Defining representative data patterns for each sensor to reduce measurement frequency.

These methods can also be combined for improved efficiency. For instance, Ref. [21] presented a simulation-based pedestrian position estimator that performs online calibration using motion detection to alternate gyroscope usage and adjust sampling rates, thereby reducing power consumption. Similarly, Ref. [22] combined adaptive sampling and duty cycling to improve energy efficiency in smartphone accelerometers.

From a different perspective, validating the effectiveness of integrating battery state of charge estimation into tracking and navigation systems, Ref. [23] proposed energy-aware tracking systems integrating photovoltaic energy harvesting and batteries. They employed closed-loop SOC estimation and statistical conflation. Their methods estimate instantaneous available energy with errors within 10%, based solely on terminal voltage and current, which reduces computational cost and enables predictive energy-aware task optimization.

Based on the literature review, a gap persists in directly integrating dynamic battery energy perception and monitoring capabilities into state estimation frameworks, particularly those for attitude determination. Unlike prior energy-efficient attitude estimation methods—which depend on external energy proxies such as motion dynamics or environmental constraints or implement energy management decoupled from the core estimator (e.g., Ref. [23])—the proposed approach directly integrates the battery’s state of charge into the Kalman filter’s state vector. This integration embeds energy awareness intrinsically within the estimation framework.

By embedding the battery State of Charge directly into the estimation framework, the proposed approach enables real-time adaptation of estimation-related operational parameters, such as sensor sampling rates, filter update frequency, and computational load allocation, according to the available energy. This adaptive behavior improves system reliability by preventing abrupt estimation degradation caused by unexpected energy depletion, enhances robustness by maintaining stable estimation performance under varying power conditions, and extends operational autonomy by allowing the system to trade estimation fidelity for energy efficiency when battery resources become constrained.

Under these circumstances, this paper proposes a Battery-Aware Extended Kalman Filter (B-EKF) that jointly addresses state of charge estimation and attitude determination within a unified framework. This integration enables the system to ascertain the available energy in its power supply concurrently with attitude determination, via an adapted SOC estimator embedded within the motion parameter estimator. Consequently, this method will permit motion detection systems to adapt their sensing and processing behavior not only to dynamic mission complexity but also in response to available energy, thereby enhancing overall system efficiency—an essential capability for modern embedded and mobile platforms.

The main contributions of this work are:

- Unlike conventional SOC estimators designed as standalone modules, the proposed SOC-EKF is formulated to share structural assumptions, timing, and covariance propagation with the attitude estimator, which is a prerequisite for the unified Battery-Aware EKF proposed in this research;
- In contrast to existing energy-aware attitude estimation approaches, where energy metrics are computed externally and used to adjust estimator parameters, the proposed framework embeds SOC directly into the Kalman filter state vector, resulting in a fully coupled and unified estimation architecture;
- It is demonstrated that, for energy-constrained motion monitoring systems operating with a single battery and within a predefined safe SOC range, the proposed B-EKF can provide sufficient real-time energy awareness to support attitude estimation and energy-aware operation without relying on a dedicated Battery Management System.

## 2. Derivation of the Coupled SOC and Attitude Kalman Filter

More recent SOC estimation approaches, such as those in Refs. [24,25,26], employ data-driven and machine learning techniques that typically achieve SOC estimation errors below 5%. However, these methods require high-dimensional models, iterative optimization, or neural network inference, resulting in increased computational complexity and memory usage, which limits their applicability in low-power wearable and embedded motion monitoring systems. Given the objective of integrating SOC into a unified framework, minimizing processing requirements is essential. To design a software-based energy estimation mechanism with reduced computational complexity, the Extended Kalman Filter was adopted. This approach ensures consistent alignment between the equations, enabling the simultaneous estimation of two distinct variables: attitude and State of Charge. For attitude estimation, a Simplified EKF for MIMU attitude estimation (SEKF) with reduced Kalman gain computation is employed to minimize processing overhead without compromising accuracy [27]. Complementarily, the implemented SOC estimator uses a one-dimensional EKF based on voltage and current measurements at the battery terminals under specific conditions. Please refer to the Appendix A for the definition of the variables presented in the formulation.

### 2.1. One-Dimensional State of Charge EKF Estimator

The State of Charge Extended Kalman Filter (SOC-EKF), expressed in (1), is configured to perform the state prediction step based on the control input term of the EKF equations. It utilizes the load current as the input variable u(t) and assumes a unitary state transition, in accordance with the dynamic relationships defined by Peukert’s law [13] and battery lifetime models [28].

(1) $$SOC_{est}=SOC_{o}-\frac{1}{C_{n}}\int_{0}^{t}I_{b}dt,$$

where

$C_{n}$ denotes the effective battery capacity.

$I_{b}$ is the battery current.

$SOC_{o}$ represents the initial state of charge.

In the update step—an intrinsic stage of Kalman Filter procedure calculated by updated state of charge (3) and updated error covariance (4)—battery discharge voltage curves were used to refine the SOC value estimated from the current measurement. Based on this data, a numerical approximation of the voltage-SOC relationship was derived to formulate the nonlinear terminal voltage equation *h*, which was then employed to compute the Kalman gain (5), the predicted state of charge (6), and the predicted error covariance (7), a statistical measure that quantifies the uncertainty of the filter’s state estimates.

(2) $$h=Ax^{3}-Bx^{2}+Cx+D$$

Parameters A, B, C, and D are derived from the numerical approximation, as specified in Equations (37) and (38) of Section 3.2.

By incorporating the covariance error extrapolation equation from the state prediction step, the SOC-EKF algorithm can be expressed as follows:

(3) $${x_{n}}^{SOC}=\;F\cdot x_{n-1}^{SOC}+G\cdot u_{n-1}^{i}$$

(4) $$P_{n}=P_{n-1}\cdot F^{2}+Q$$

(5) $$K_{n}=\frac{H\cdot P_{n}}{P_{n}\cdot H^{2}+R}$$

(6) $${x_{n+1}}^{SOC}={x_{n}}^{SOC}+K_{n}\cdot \left(z_{n}^{v}-H\right)$$

(7) $$P_{n+1}={\left(1-K_{n}\cdot H\right)}^{2}\cdot P_{n}$$

Here,

F=1 represents the unitary transition element.
$G=-\Delta t/C_{n}$ is the control element.
$C_{n}$ is the battery capacity satisfying the SOC discharge dynamic model.
$R=\sigma_{v}$ is the measurement noise.
$Q=\sigma_{i}G^{2}$ denotes the process noise projection, according to the continuous control matrix model [29].
$x_{n}^{SOC}$ indicates the SOC estimate.
$u_{n}^{i}$ is the input current.
$z_{n}^{v}$ corresponds to the measured battery terminal voltage.

The linearized observer element *H*, derived from the Taylor Series expansion of *h*, is given by $H=h_{x_{o}}+\left(3Ax^{2}-2Bx+C\right)\left(x-x_{o}\right)$. The Kalman gain is denoted by $K_{n}$, while $P_{n}$ is the state error covariance, and $\sigma_{v}$ and $\sigma_{i}$ represent the variance of the battery’s voltage and current measurements, respectively.

It is important to emphasize that the proposed SOC-EKF is not intended as a standalone battery estimator. Instead, its structure, dimensionality, and covariance propagation are deliberately defined to enable direct mathematical coupling with the attitude estimation process described in Section 2.3.

Due to these simplifications, a slight reduction in SOC estimation accuracy is expected. However, given the focus on minimizing computational load and motivated by Ref. [23] results, this trade-off was considered attainable.

### 2.2. Attitude Estimator

Although the EKF is widely used with IMUs, its high computational cost remains a major limitation. Detailed analyses have shown that the main source of this cost is the matrix inversion required for the Kalman gain computation [30].

In this context, incorporating SOC-EKF estimation with the SEKF proposed by Ref. [27] was adopted as it eliminates the matrix inversion in the Kalman gain calculation, preserving acceptable estimation accuracy while reducing the algorithm’s execution time by approximately 47% compared to the standard EKF implementation.

This integration was possible considering the following conditions:

- The MIMU sampling frequency satisfies $f_{s}>>1$;
- Only small-amplitude rotational motions occur;
- The gyroscope variance satisfies $\sigma_{\omega }^{2}<<1$;
- The initial estimates of the attitude quaternion $q_{0}$ and the error covariance matrix $P_{0}$ are provided by the QUEST algorithm, a classic and highly efficient method for estimating the attitude of a rigid body from vector measurements, used in Takahashi [31].

The SEKF equations adapted for this work are presented below. These equations rely on the construction of mutually orthogonal observation vectors derived from accelerometer and magnetometer measurements.

(8) $$b_{1}=g_{b}$$

(9) $$b_{2}=\left(m_{b}-g_{b}cos\left(\theta_{b}\right)\right)/sin\left(\theta_{b}\right)$$

(10) $$z_{n}=\left[\begin{array}{c} b_{1}\\ b_{2} \end{array}\right]$$

Here, $g_{b}$ and $m_{b}$ denote the gravitational acceleration and geomagnetic field unitary vectors, respectively. $b_{1}$ and $b_{2}$ are observer vectors and $z_{n}$ is the measurement vector. The skew-symmetric matrix ($\Omega_{n}$) of the gyroscope angular velocity measurements (12) is used in the prediction stage, where the State Transition Matrix $\Phi_{n}$ is computed as in (11).

(11) $$\Phi_{n}=cos\left(\frac{\left|\omega_{n}\right|}{2f_{s}}\right)I_{4\times 4}+\frac{sin\left(\frac{\left|\omega_{n}\right|}{2f_{s}}\right)}{\left|\omega_{n}\right|}\Omega_{n}$$

(12) $$\Omega_{n}=\left[\begin{array}{cccc} 0 & \omega_{3} & -\omega_{2} & \omega_{1}\\ -\omega_{3} & 0 & \omega_{1} & \omega_{2}\\ \omega_{2} & -\omega_{1} & 0 & \omega_{3}\\ -\omega_{1} & -\omega_{2} & -\omega_{3} & 0 \end{array}\right]$$

The vector $\omega_{n}={\left[\omega_{1}\omega_{2}\omega_{3}\right]}^{T}$ represents the three-dimensional gyroscope rate. From this matrix, the predicted attitude quaternion values $q_{n+1/n}$ are obtained using (13).

(13) $$q_{n+1/n}=\Phi_{n}\cdot q_{n}$$

In the SEKF update step, the reference observation vectors are defined as $r_{1}={\left[0;0;-1\right]}^{T}$ and $r_{2}={\left[1;0;0\right]}^{T}$, corresponding to the North-East-Down (NED) coordinate frame. These vectors are combined with the estimated quaternion $q_{n+1}$ and the Direction Cosine Matrix (DCM) $C_{n}^{b}$ to construct the observation matrix ψ. Due to its nonlinear nature, this matrix is linearized using the Jacobian approach, resulting in matrix Ψ.

Next, the values of the simplification parameters (*a*, *b*, *c*, and *d*) and matrices (**B**, **V**, and **M**) are updated. These quantities are derived from the magnetometer and accelerometer variances and are used to compute the Kalman Gain without matrix inversion (14), as well as the quaternion update (15) and error covariance matrix (16) equations.

(14) $$K_{n+1}^{s}=\frac{a}{4bd\left(bd-a^{2}\right)}\left(\Psi_{n+1}^{T}+\frac{4c}{a}q_{n+1/n}V_{n+1}^{T}\right)M_{n+1}$$

(15) $$q_{n+1}=q_{n+1/n}+K_{n}^{s}\left(z_{n}-\psi \left(q_{n+1/n}\right)\right)$$

(16) $$P_{n+1/n+1}^{s}=\left(\alpha_{n}^{2}+\frac{c}{4}\right)\left[1-a\frac{b+d}{bd}\right]I_{4\times 4}$$

Finally, the recursive computation of $\alpha_{n}^{2}$ in the SEKF is performed using (17), which updates the simplification parameters and matrices for the next iteration.

(17) $$\alpha_{n+1}^{2}=\left(\alpha_{n}^{2}+\frac{c}{4}\right)\left[1-a\frac{b+d}{bd}\right]$$

### 2.3. Coupling of Attitude and SOC Estimation

Leveraging the similar structure between SEKF and SOC-EKF, it is feasible to merge these two filters through appropriate mathematical restructuring. This involves expanding the state vectors and using a composite observation matrix Ψ, adaptable to measurement availability. This integration begins by characterizing the SEKF conditions and its a posteriori attitude estimation error covariance matrix:

(18) $$P_{n/n}\approx \alpha_{n+1}^{2}I_{4x4}\approx \Phi_{n}P_{n/n}\Phi_{n}^{T}$$

If $f_{s}>>\left|\omega_{n}\right|$ and F=1, the following approximation holds true:

(19) $$\Phi_{n}^{SOC}\approx I_{5\times 5}$$

Then,

(20) $$P_{n/n}^{SOC}\approx \left[\begin{array}{ccc} \alpha_{n+1}^{2}I_{4\times 4} & \; & 0_{4\times 1}\\ 0_{1\times 4} & \; & P_{n} \end{array}\right]$$

Thus, the expansion of the B-EKF State Transition Matrix ($\Phi_{n}^{SOC}$) and the B-EKF estimation error covariance matrix ($P_{n/n}^{SOC}$) can be performed without modifying the parameters or inherent characteristics of the estimator. Consequently, the State Extrapolation Equation is extended to incorporate the SOC state and include the current control input term, as presented in Equation (21).

(21) $$\left[\begin{array}{c} q_{n+1}\\ x_{n+1}^{SOC} \end{array}\right]=\left[\begin{array}{cc} \Phi_{n} & 0_{4\times 1}\\ 0_{1\times 4} & F \end{array}\right]\cdot \left[\begin{array}{c} q_{n}\\ x_{n}^{SOC} \end{array}\right]+\left[\begin{array}{c} 0_{4\times 1}\\ G \end{array}\right]\cdot u_{n-1}^{i}$$

The SEKF Gaussian noise covariance matrix is derived from (22), through gyroscope rate variance $\sigma_{\omega }$. $A_{n}$ was defined by Ref. [27].

(22) $$Q_{n}={\left(\frac{\sigma_{\omega }}{2f_{s}}\right)}^{2}\left(I_{4\times 4}-A_{n}\right)=kI_{4\times 4}-kA_{n}$$

By concatenating the SEKF covariance matrix equations with the SOC covariance element, the combined formulation is obtained:

(23) $$Q_{n}^{SOC}=\left[\begin{array}{ccc} {\left(\frac{\sigma_{\omega }}{2f_{s}}\right)}^{2}\left(I_{4\times 4}-A_{n}\right) & \; & 0_{4\times 1}\\ 0_{1\times 4} & \; & \sigma_{i}.G^{2} \end{array}\right]$$

The same expansion applies to the simplification matrices V and M, as shown in (24) and (25).

(24) $$V_{n}^{SOC}=\left[\begin{array}{c} V\\ 0 \end{array}\right]$$

(25) $$M_{n}^{SOC}=\left[\begin{array}{cc} M & 0_{6\times 1}\\ 0_{1\times 6} & K_{1} \end{array}\right]$$

Here, $K_{1}=H\cdot P_{n}$ represents a term derived from the Kalman gain equation of the SOC estimator, reformulated to match the structure of the $K_{n}^{SOC}$ equation.

The observation matrix is expanded by incorporating the term $K_{2}=1/\left(H^{2}\cdot P_{n}+R\right)$, which also originates from the SOC estimator’s gain equation. The resulting matrix $H_{n}^{SOC}$ is expressed as (26).

(26) $$H_{n}^{SOC}=\left[\begin{array}{cc} \Psi & 0_{4\times 1}\\ 0_{1\times 6} & K_{2} \end{array}\right]$$

Since the SEKF uses the predicted quaternion $q_{n+1/n}$ to update the gain value, whereas the SOC estimator does not, an expansion with a null element is introduced in this estimation step, yielding $q_{K}={\left[q_{n+1/n}\;0\right]}^{T}$.

Consequently, the Kalman gain equation for the coupled estimator (attitude and SOC) is defined as follows:

(27) $$K_{n+1}^{SOC}=\frac{a}{4bd\left(bd-a^{2}\right)}\left(H_{n+1}^{SOC^{T}}+\frac{4c}{a}q_{k}V_{n+1}^{SOC^{T}}\right)M_{n+1}^{SOC}$$

The attitude and state of charge values are then computed using (28), where $K_{n+1}^{SOC}$, the nonlinear observation matrix $h^{SOC}$, and the measurements from the accelerometer, magnetometer, and battery terminal voltage are combined.

(28) $$q_{n+1}^{SOC}=q_{n+1/n}^{SOC}+K_{n+1}^{SOC}\left(z_{n}^{SOC}-h^{SOC}\left(q_{n+1/n}^{SOC}\right)\right)$$

Given that $q_{n+1/n}^{SOC}={\left[q_{n}\;x_{n}^{SOC}\right]}^{T}$, the measurement vector $z_{n}^{SOC}$ and the nonlinear observation function $h_{n}^{SOC}$ are defined as:

(29) $$z_{n}^{SOC}=\left[\begin{array}{c} z_{n}\\ z_{n}^{v} \end{array}\right]$$

(30) $$h_{n}^{SOC}=\left[\begin{array}{c} \psi \\ h \end{array}\right]$$

Finally, the estimation error covariance matrix is updated, approximated according to the relationship previously established in (20). Based on this formulation, each estimation process is performed independently within the Kalman filter architecture while maintaining the mathematical coupling between attitude determination and battery SOC estimation during system operation.

**Figure 1:**
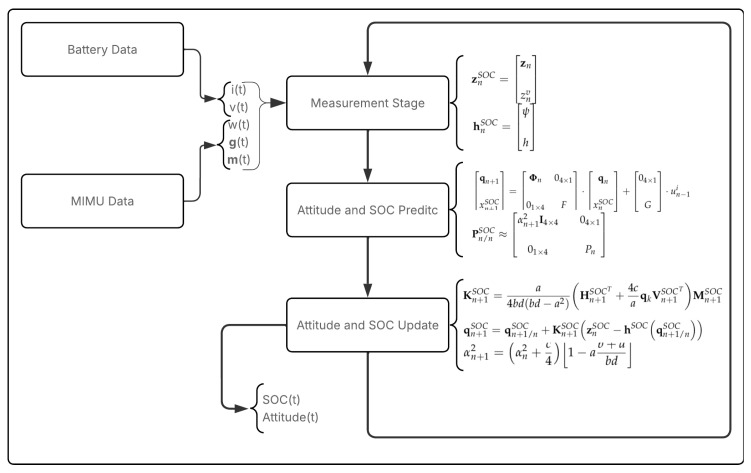
B-EKF Flowchart.

The formulation of algorithm developed to execute each step of the Battery-Aware Extended Kalman Filter is presented by Equations (17), (20) and (21), at Predict Step, (27) and (28), at Update Step, and (29) and (30) at Measurement Step, as depicted in the flowchart in Figure 1, where it can also be noted: the input signals—current i(t), terminal voltage v(t), angular velocity w(t), gravitational field g(t), and geomagnetic field m(t)—are computationally generated to validate the proposed methodology. These data are then processed by the B-EKF to estimate the attitude of the system while simultaneously assessing the available energy through the battery’s state of charge.

This formulation differs fundamentally from energy-aware estimation strategies reported in the literature, as the SOC is estimated and propagated as an internal state of the filter, rather than being treated as an external input or supervisory variable.

## 3. Experimental Results and Discussions

The performance of the proposed B-EKF was validated through a series of simulations designed to verify the maintenance of original SEKF attitude accuracy proposed by Rong et al. [27], the SOC-EKF adequate accuracy and implementation feasibility compared with the results produced by Sommer et al. [23].

### 3.1. Attitude Data Generation

The simulated measurements for the gyroscope ($\bar{\omega_{n}}$), accelerometer ($g_{b}$), and magnetometer ($m_{b}$) were defined as follows:

(31) $$\bar{\omega_{n}}=\omega_{n}+v_{n}^{\omega }=\left[\begin{array}{c} \omega_{nx}\\ \omega_{ny}\\ \omega_{nz} \end{array}\right]+v_{n}^{\omega }=\left\{\begin{array}{c} 3sin\left(3\pi \frac{n-1}{F_{s}}\right)\\ 4sin\left(4\pi \frac{n-1}{F_{s}}\right)\\ 5sin\left(5\pi \frac{n-1}{F_{s}}\right) \end{array}\right.+v_{n}^{\omega }$$

(32) $$g_{b}=C_{n}^{b}\left(\bar{\omega_{n}}\right)g_{r}+v_{n}^{a}$$

(33) $$m_{b}=C_{n}^{b}\left(\bar{\omega_{n}}\right)m_{r}+v_{n}^{m}$$

where $g_{r}$ and $m_{r}$ represent the reference values of the accelerometer and magnetometer, respectively, at sampling instant *n*; the $F_{s}$ is sample frequency; $v_{n}^{w}$, $v_{n}^{a}$, and $v_{n}^{m}$ denote the white Gaussian measurement noise components. The rotation matrix $C_{n}^{b}\left(\bar{\omega_{n}}\right)$ is iteratively updated from its initial value $C_{n}^{b}\left(0\right)$, as proposed by Rong et al. [27]:

(34) $$C_{n}^{b}\left(0\right)=\left[\begin{array}{ccc} -0.8957 & 0.1560 & 0.0637\\ -0.1203 & -0.9161 & 0.3825\\ 0.1181 & 0.3694 & 0.9217 \end{array}\right]$$

This update is performed using the Poisson Kinematical Equation expressed in (35) and the composite trapezoidal integration method [32]:

(35) $$\dot{C_{n}^{b}}={℧}^{b}C_{n}^{b}$$

(36) $${℧}^{b}=\left[\begin{array}{ccc} 0 & -\omega_{3} & \omega_{2}\\ \omega_{3} & 0 & -\omega_{1}\\ -\omega_{2} & \omega_{1} & 0 \end{array}\right]$$

**Figure 2:**
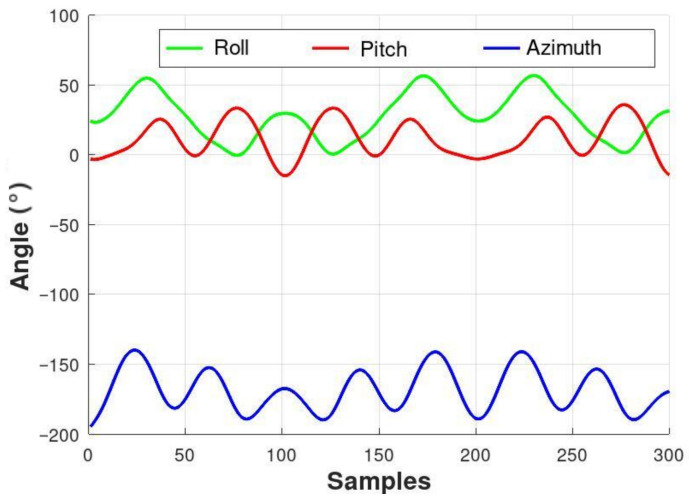
Emulated time history of attitude reference.

The initial attitude estimate $q_{o}=$ [0.0495 0.2121 0.9661 0.1380] was obtained using the QUEST algorithm described in Takahashi [31]. The simulated data variance for the gyroscope was set to $\sigma_{\omega }^{2}=0.06^{2}$, as disigned by Rong et al. [27], and subsequently applied to the accelerometer and magnetometer. The time history of the reference attitude obtained from this data is presented in Figure 2.

### 3.2. SOC Data Generation

**Figure 3:**
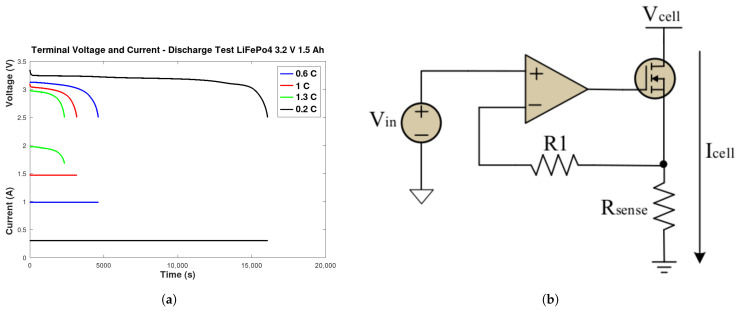
Complete battery discharge cycle test. (**a**) Terminal Voltage and Current of All loads. (**b**) Active electronic load circuit.

Consumption data were generated using voltage, current, and SOC linear curves acquired from an active electronic load circuit (Figure 3b), performing a complete battery discharge cycle test (Figure 3a).This setup simulated a specific SOC profile device, as verified by [33,34], connected to a LiFePO4 battery rated at 3.2 V and 1500 mAh, similar to [17], operating at 0.2 C, 0.6 C, 1 C, and 1.3 C.

**Figure 4:**
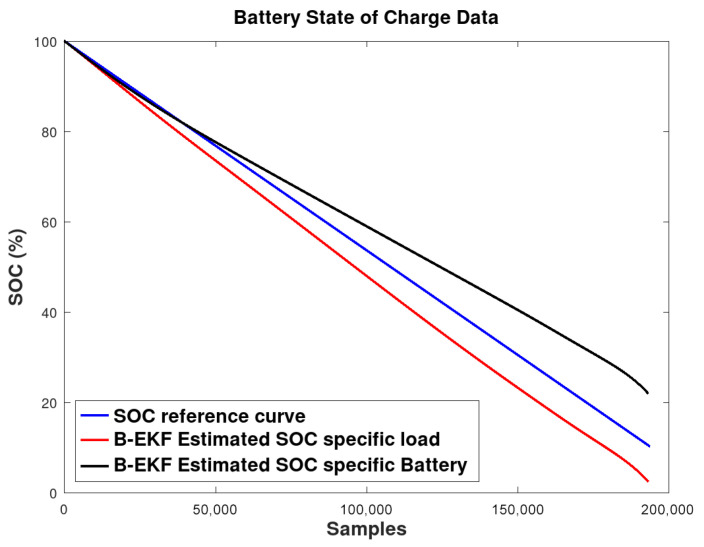
Reference SOC and estimated SOC by B-EKF for specific load and specific battery scenarios.

A reference SOC curve ( Figure 4), generated from complete battery discharge cycle test, was then used to evaluate the accuracy of the SOC estimation under two distinct scenarios:

- **Scenario 1**—SOC estimation for a single battery supplying a specific load;
- **Scenario 2**—SOC estimation for a specific battery operating under various load conditions.

**Figure 5:**
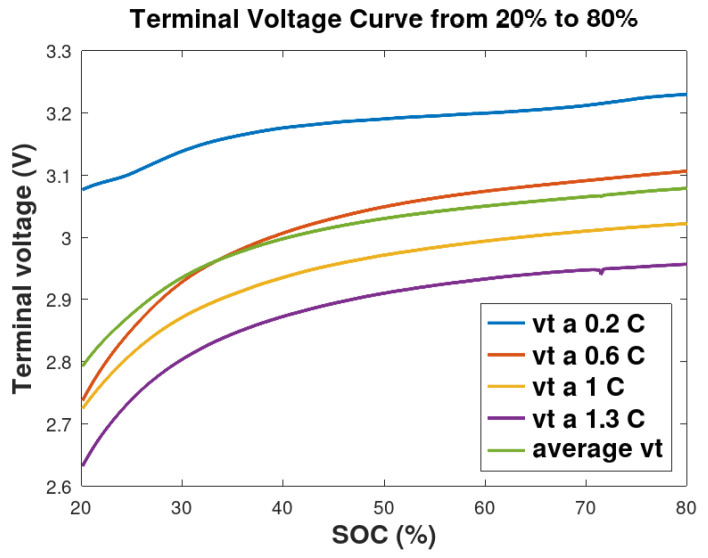
Battery discharge voltage curves from 20% to 80% state of charge at discharge rates of 2.0, 0.6, 1.0 and 1.5 C along with the corresponding average discharge voltage curve.

Two observation equations, denoted $h_{1}$ and $h_{2}$, were established. Referring to Figure 5, the first, $h_{1}$, models the terminal voltage under a specific 0.2 C discharge load (Scenario 1, blue curve). The second, $h_{2}$, is based on the averaged terminal voltage across all voltage discharge tests (Scenario 2, green curve). Battery temperature effects were not considered in this derivation. Thus, $h_{1}$ captures a particular load condition, whereas $h_{2}$ represents the battery’s typical operation for different loads. The other curves represent the battery discharge voltage under their respective load conditions: 0.6 C (red curve), 1 C (yellow curve), and 1.3 C (purple curve). These curves were limited to SOC values ranging from 20% to 80%, as can be seen in Figure 5, corresponding to the safety battery’s operational range.

(37) $$h_{1}=1.8579x^{3}-3.223x^{2}+1.9388x+2.796$$

(38) $$h_{2}=2.7423x^{3}-5.0816x^{2}+3.2466x+2.3383$$

The variance values used were $\sigma_{i}^{2}=0.01$, obtained from the measurement instrument’s datasheet, and $\sigma_{v}^{2}=0.0289$, calculated based on the worst-case deviation between the experimental discharge voltage curves and their average discharge voltage curve.

### 3.3. Experimental Results

To perform the validation tests for the B-EKF, the attitude reference time histories presented in Section 3.1 were used alongside the SOC data from the 0.3 C curve, around 0.1 W power consumption, from a full battery cycle experiment detailed in Section 3.2. This profile was selected to minimize the effects of battery heating on the results and to represent the typical power consumption range of wearable systems, from 0.02 to 0.1 W [19].

The attitude data were generated using the GNU OCTAVE programming language, within the same environment where experimental data for terminal voltage, current, and battery state of charge were organized. The implementation of B-EKF (in the C programming language) was carried out through a total of 645 SEKF runs, each consisting of 300 samples, alongside a single SOC-EKF run comprising 193,574 samples, corresponding to a full battery discharge cycle. A sampling rate of 100 Hz was selected and found to be sufficient to capture the dynamic behavior of both attitude and SOC.

#### 3.3.1. Attitude Estimation

**Figure 6:**
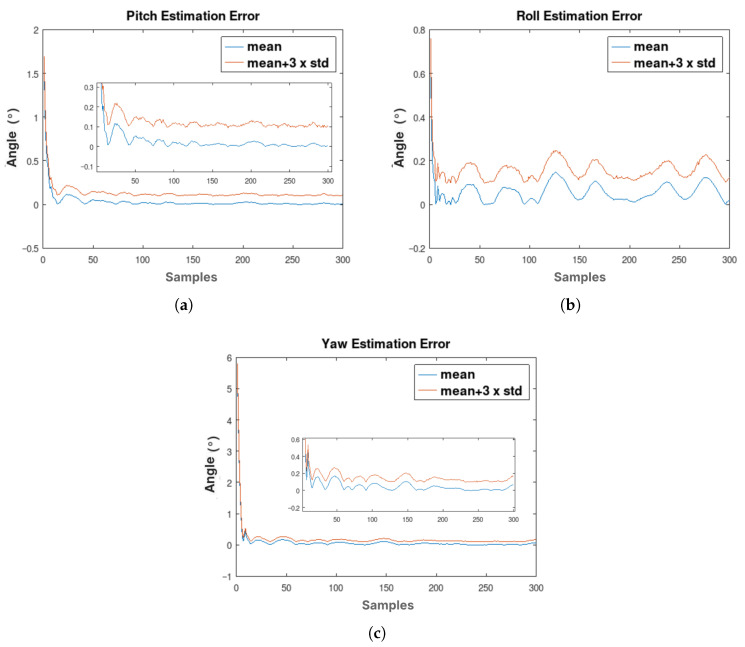
Mean of absolute error variation of 645 iterations. (**a**) Changes in pitch estimation error. (**b**) Changes in roll estimation error. (**c**) Changes in yaw (azimuth) estimation error.

The time histories of the mean and standard deviation (3σ) of the absolute attitude estimation errors across all trials are presented in Figure 6.

The roll, pitch, and yaw (azimuth) estimation results show a reduction in error values at the beginning of the estimation process and subsequently stabilize, displaying random behavior due to the introduction of noise into the data derived from Equations (31)–(33), as well as to the errors and uncertainties inherent in the B-EKF estimates. As expected, the statistical characteristics of the attitude estimation errors obtained with the B-EKF are consistent with the accuracy demonstrated by the original SEKF from Rong et al. [27], remaining around 0.2° despite the inclusion of the additional computations required for battery SOC estimation.

#### 3.3.2. Energy Estimation

**Figure 7:**
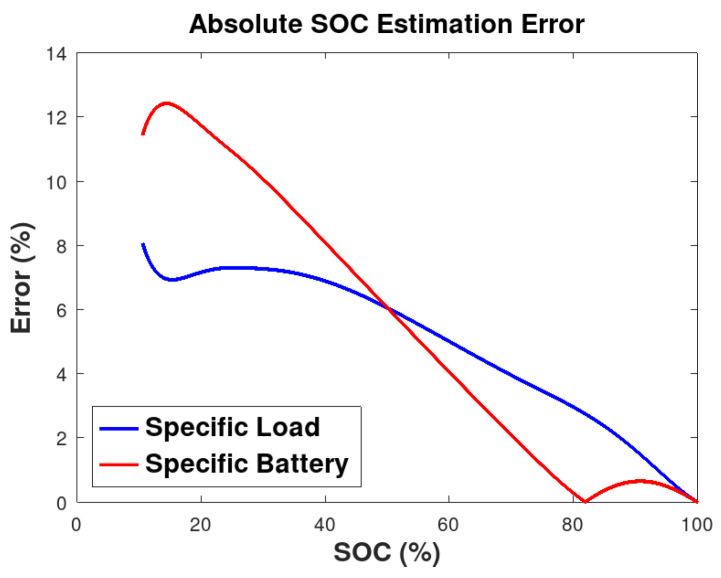
Absolute error of SOC estimation for specific load and specific battery scenarios.

The absolute error of the SOC estimation is illustrated in Figure 7. Under Scenario I conditions, the results indicate adequate estimation accuracy during the fully charged stage, with the error increasing to approximately 8% as the battery approaches full discharge. In scenario II, where a single battery is evaluated while supplying different loads, the estimation error rises around to 12% near the discharge limit, whereas errors below 6% are observed for partially discharged states (SOC > 50%).

These results demonstrate that the B-EKF adequately estimates SOC in both scenarios, improving upon the decoupled method in Sommer et al. [23], which reported errors of up to 10%. This improved performance remained consistent over the full discharge cycle.

#### 3.3.3. Computing Resources

Considering that the applications targeted by the proposed B-EKF typically rely on battery-powered embedded platforms, such as microcontroller-based systems or low-power System-on-Chip (SoC) architectures, the algorithm was designed under embedded implementation constraints and implemented in standard C to ensure portability. Validation was carried out on an Intel^®^ Core™ i5 processor running Debian 12. Using the standard time.h library, a preliminary computational cost analysis was performed by executing 1000 independent iterations of the SEKF, SOC-EKF, and B-EKF algorithms. The average elapsed time per iteration was then computed to enable a comparison between the sequential execution of the two independent filters and the unified execution of the proposed method.

The B-EKF exhibited a slight increase in execution time against to the SEKF, on the order of $2\times 10^{-6}$ s, attributed to the additional SOC estimation operations integrated into the implementation. This overhead is negligible when compared to the $134.2\times 10^{-6}$ s required for the standard SEKF execution and when considering the gains provided by the added battery energy monitoring capability. The comparison between the B-EKF and the sequential execution of the separate SEKF and SOC-EKF (without integration) demonstrates the distinct advantages of the B-EKF, include a reduction in memory usage, achieved by eliminating the need for additional variables; a decrease of 21.08% in processing time ($28.3\times 10^{-6}$ s less than the sequential execution, on average); and a lower number of required iterations compared to the separate execution of the two filters.

## 4. Conclusions

In this article, a one-dimensional State of Charge Extended Kalman Filter was developed to operate exclusively with battery terminal voltage and current input data. This simplification was designed to reduce computational demands and enable the algorithm’s adaptability for attitude determination in systems with critical energy resources. Based on this design, rather than proposing an isolated novel SOC estimation algorithm, this work demonstrates how a computationally efficient SOC-EKF can be reformulated and embedded within an attitude estimation framework. This integration enables energy-aware sensor fusion without increasing estimator complexity, culminating in the development of the Battery-Aware Extended Kalman Filter.

The proposed method is not intended to improve attitude estimation and carries all the limitations inherent to validation tests performed under simulation conditions. Nevertheless, the B-EKF achieves accuracy equivalent to that of the original SEKF presented by Rong et al. [27] with negligible additional computational cost. Furthermore, the developed one-dimensional SOC-EKF integrates effectively into the SEKF structure and produces estimates that are sufficiently accurate for orientation and attitude determination in energy-constrained systems.

The Battery-Aware Extended Kalman Filter departs from prior energy-aware sensor fusion architectures by embedding battery energy estimation directly into the state estimation process, resulting in a unified and mathematically coupled framework for MIMU attitude determination. Once validated in future embedded motion monitoring systems subject to energy constraints, the proposed method will provide a lightweight alternative to standalone SOC estimation modules, enabling energy-aware attitude estimation for systems with limited energy resources and modest battery management requirements. This capability allows such systems to dynamically adapt their operational behavior by integrating motion dynamics, environmental constraints, and the energy available for task execution.

## Data Availability

Access to the data generated in this research is subject to a formal request to the authors.

## Abbreviations

The following abbreviations are used in this manuscript:

EKF	Extended Kalman Filter
MIMU	Magnetic and Inertial Measurement Unit
IMU	Inertial Measurement Unit
SOC	State of Charge
SEKF	Simplified EKF for MIMU attitude determination
BMS	Battery Management System
MEMS	Micro-Electro-Mechanical System
DCM	Direction Cosine Matrix
QUEST	Quaternion estimator algorithm
SOC-EKF	State of Charge Extended Kalman Filter
B-EKF	Battery-Aware Extended Kalman Filter

## Appendix A

$C_{n}$	Effective battery capacity
$I_{b}$	Battery current
$SOC_{o}$	Initial state of charge
*F*	SOC-EKF transition element
*G*	SOC-EKF control element
*R*	SOC-EKF measurement noise
*Q*	SOC-EKF process noise
$K_{n}$	SOC-EKF kalman gain
$P_{n}$	SOC-EKF state error covariance
$x_{n}^{SOC}$	SOC estimate
$u_{n}^{i}$	Input current
$z_{n}^{v}$	Measured battery terminal voltage
*h*	Nonlinear terminal voltage equation
*H*	Linearized observer element
$\sigma_{v}$	Variance of terminal voltage
$\sigma_{i}$	Variance of terminal current
$g_{b}$	Gravitational acceleration of the body
$m_{b}$	Geomagnetic field of the body
$b_{1}$	Observer vector 1
$b_{2}$	Observer vector 2
$z_{n}$	Measurement vector
$\Phi_{n}$	SEHK state transition matrix
$\Omega_{n}$	Skew-symmetric matrix
$q_{n+1/n}$	Predicted attitude quaternion
$q_{n}$	Current attitude quaternion
$q_{n+1}$	Updated attitude quaternion
$r_{1}$	Reference observation vector 1
$r_{2}$	Reference observation vector 2
$C_{n}^{b}$	Direction cosine matrix (DCM)
ψ	SEKF nonlinear observation matrix
Ψ	SEKF linearized observation matrix
$K_{n+1}^{s}$	SEKF kalman gain
$P_{n+1/n+1}^{s}$	SEKF error covariance matrix
$\Phi_{n}^{SOC}$	B-EKF state transition matrix
$P_{n/n}^{SOC}$	B-EKF error covariance matrix
$K_{n+1}^{SOC}$	B-EKF kalman gain
$q_{n+1}^{SOC}$	B-EKF updated attitude quaternion SOC
